# Identification of novel genes associated with HIV-1 latency by analysis of histone modifications

**DOI:** 10.1186/s40246-017-0105-7

**Published:** 2017-05-12

**Authors:** Kyung-Chang Kim, Sunyoung Lee, Junseock Son, Younghyun Shin, Cheol-Hee Yoon, Chun Kang, Byeong-Sun Choi

**Affiliations:** Division of AIDS, Center for Immunology and Pathology, Korea National Institute of Health, Chung-buk, 28160 Republic of Korea

**Keywords:** HIV latency, Histone modification, Chromosome, H3K4me3, H3K9ac

## Abstract

**Background:**

A reservoir of HIV-1 is a major obstacle in eliminating HIV-1 in patients because it can reactivate in stopping antiretroviral therapy (ART). Histone modifications, such as acetylation and methylation, play a critical role in the organization of chromatin domains and the up- or downregulation of gene expression. Although many studies have reported that an epigenetic mechanism is strongly involved in the maintenance of HIV-1 transcriptional latency, neither the epigenetic control of viral replication nor how HIV-1 latency is maintained is not fully understood.

**Results:**

We re-analyzed a high throughput parallel DNA sequencing (ChIP-seq) data from previous work to investigate the effect of histone modifications, H3K4me3 and H3K9ac, on HIV-1 latency in terms of chromosome distribution. The outputs of ChIP-seq from uninfected CD4+ T cell lines and HIV-1 latently infected cells were aligned to hg18 using bowtie and then analyzed using various software packages. Certain chromosomes (16, 17, 19, and 22) were significantly enriched for histone modifications in both decreased and increased islands. In the same chromosomes in HIV-1 latently infected cells, 38 decreased and 41 increased islands from common islands of H3K4me3 and H3K9ac were selected for functional annotation. In Gene Ontology analysis, the 38 genes associated with decreased islands were involved in the regulation of biological process, regulation of cellular process, biological regulation, and purinergic receptor signaling pathway, while the 41 genes associated with increased islands were involved in nucleic acid binding, calcium-activated cation channel activity, DNA binding, and zinc ion binding. In Pathway Commons analysis, the 38 genes were strongly involved in the p63 transcription factor network, while the 41 genes were involved in the RNA polymerase III transcription termination pathway. Several genes such as Nuclear factor I X (*NFIX*) and TNF receptor association factor 4 (*TRAF4*) were selected as candidate genes for HIV latency. Especially, *NFIX* was highly expressed in HIV-1 latently infected cell lines and showed a dramatic reduction in expression after phorbol-13-myristate-12-acetate (PMA) treatment.

**Conclusions:**

These results show that the unique enrichment of histone modifications and its linked genes in specific chromosomes might play a critical role in the establishment and maintenance of HIV-1 latency.

**Electronic supplementary material:**

The online version of this article (doi:10.1186/s40246-017-0105-7) contains supplementary material, which is available to authorized users.

## Background

HIV-1 can establish a state of latency in the early stages of infection. The integration of viral cDNA into host chromosomes is a fundamental step, after which, viral genomes can persist for the lifespan of infected cell [1]. Despite combination antiretroviral therapy (cART), which can repress HIV-1 replication and delay the progression of AIDS, HIV-1 reemerges rapidly after an interruption in treatment [2, 3]. Due to the long half-life of viral reservoirs, it has been reported that the eradication of HIV-1 with current drugs would require over 60 years [4]. The transcription of HIV-1 genome depends on both viral and cellular factors [5]. The activity of HIV promoter is closely connected to the activation of host cells [6]. Additionally, gene expression of HIV-1 latency can be dynamically regulated through epigenetic changes in chromatin structure surrounding and within integrated HIV-1 provirus [7, 8]. In particular, acetylation and methylation have considered as critical elements of chromatin activity and play a critical role in gene regulation [9]. Recently, Park et al. [10] generated the genome-wide maps of histone modifications for histone 3 lysine 4 trimethylated (H3K4me3) and histone 3 lysine 9 acetylated (H3K9ac) using HIV-1 latently infected cells and uninfected cells. They found that histone modification near HIV-1 integrated regions did not show significantly different patterns compared with control and that the enrichment of histone modification was generally no difference before and after HIV-1 integration. In addition, HIV-1 is able to establish interchromosomal interactions to control viral transcription [11]. Although many studies have reported that an epigenetic mechanism is strongly involved in the maintenance of HIV-1 transcriptional latency [12–16], neither the epigenetic control of viral replication nor how HIV-1 latency is maintained is not fully understood.

Here, we re-analyzed chromatin immunoprecipitation-high throughput parallel DNA sequencing (ChIP-seq) data from Park et al. in AIDS [10] to examine the effect of histone modifications on HIV-1 latency in terms of chromosomal distribution and to find novel genes related to HIV-1 latency.

## Results

### Chromosomal distribution of enriched histone binding sites in HIV-1 latently infected cells

**Fig. 1:**
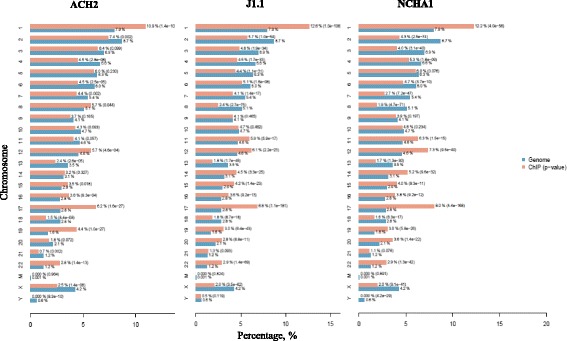
Chromosomal distribution of H3K4me3 binding sites in HIV-1 latently infected cells. Enrichment pattern of decreased H3K4me3 islands in individual chromosomes is shown as a bar chart. Percentage of total H3K4me3 islands (*red bar*) and expected results by random chance (*blue bars*) for each chromosome is shown. The *p* value is shown in *parentheses*

To identify binding sites of histone modifications such as H3K4me3 and H3K9ac, ChIP-seq data was obtained from the Gene Expression Omnibus (GEO) data repositories (GSE58246). Spatial clustering for identification of ChIP-enriched regions (SICER) 1.1 [17] was used to find differentially enriched histone islands. The number of decreased islands at H3K4me3 and H3K9ac histone modifications ranged from 1587 to 13,173 in HIV-1 latently infected cells (ACH2, NCHA1, and J1.1 cells). Increased islands ranged from 2078 to 9875 (Table 1). These results suggest that the binding sites of histone modifications may differ according to cell lines. Next, to determine the binding distribution pattern of H3K4me3 and H3K9ac across individual chromosomes, the cis-regulatory element annotation system (CEAS) software [18] was used with the decreased and increased island data (Fig. 1). Significant chromosomes were determined as those with a *p* value <10^4^, those with a ratio of histone modification binding percentage to genome background binding percentage on each chromosome >1.3 and those showing both decreased and increased islands together in all HIV-1 latently infected cells. Chromosomes 16, 17, 19, and 22 were selected by these criteria for comparison of decreased and increased islands on the same chromosome (Additional files 1, 2, 3, and 4). The results suggested that histone modifications binding to host genomes are non-random and preferentially bind to specific chromosomes.

**Table 1:** Numbers of islands identified by SICER 1.1 in HIV-1 latently infected cells according to histone modifications

Cell line	Dec or Inc	Histone modifications	
		H3K4me3	H3K9ac
ACH2	Dec	1587	3344
	Inc	2078	5563
J1.1	Dec	8634	13,173
	Inc	6941	9875
NCHA1	Dec	5275	7124
	Inc	6622	8084

*Dec* decreased islands identified by SICER 1.1, *Inc* increased islands identified by SICER 1.1, *ACH2 and J1.1* HIV-1 latently infected cells, *NCHA1* novel chronic HIV-1 infected cells derived from A3.01

### Identification of common decreased and increased islands in chromosomes 16, 17, 19, and 22

**Fig. 2:**
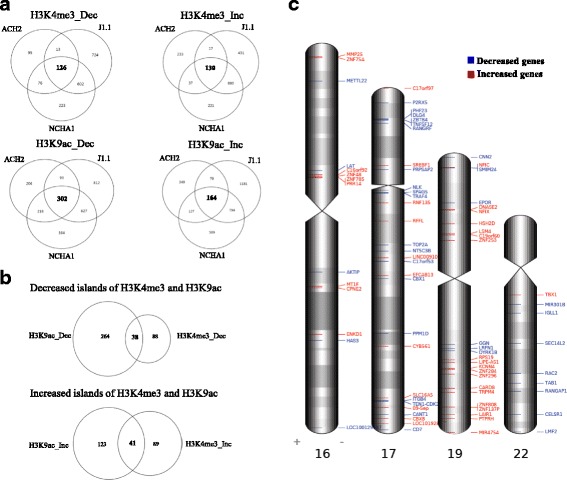
Identification of H3K4me3 and H3K9ac islands in chromosomes 16, 17, 19, and 22 of HIV-1 latently infected cells. **a** Venn diagram of differentially enriched histone modification islands in chromosomes 16, 17, 19, and 22 of HIV-1 latently infected cells. **b** The differential histone H3K4me3 and H3K9ac islands were compared and the co-occurrence of two histone modification islands was examined. **c** Human chromosome map showing the localization of 38 decreased and 41 increased genes of islands in chromosomes 16, 17, 19, and 22 of HIV-1 latently infected cells. Genes associated with decreased islands are highlighted in *blue*, and genes associated with increased islands are highlighted in *red*

We investigated common decreased and increased islands in selected chromosomes of HIV-1 latently infected cells. As a result, we identified 126 and 302 decreased islands in H3K4me3 and H3K9ac, respectively (Fig. 2a). In particular, 38 islands were identified as common decreased islands in both H3K4me3 and H3K9ac (Fig. 2b). Meanwhile, 130 and 164 increased islands were identified in H3K4me3 and H3K9ac, respectively, and 41 islands were identified as common increased islands in both H3K4me3 and H3K9ac. To find out where islands were distributed on each chromosome, the location of 38 decreased and 41 increased islands were visualized on chromosomes 16, 17, 19, and 22 using the idiographica webtool (Fig. 2c). The results suggested that HIV-1 latency may be dominated by common factors.

### Functional annotation of 38 decreased and 41 increased islands in both H3K4me3 and H3K9ac

**Fig. 3:**
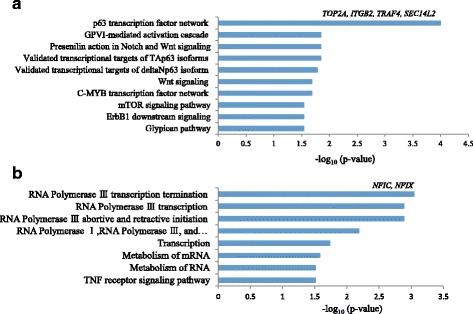
Functional annotation of 38 genes associated with decreased (**a**) and 41 genes associated with increased (**b**) islands on chromosomes 16, 17, 19, and 22 of HIV-1 latently infected cells using Pathway Commons analysis. *TOP2, ITGB2, TRAF4*, and *SEC14L2* in the 38 genes associated with decreased islands were strongly linked to the p63 transcription factor network. Meanwhile, *NFIC* and *NFIX* in the 41 genes associated with increased islands were related to RNA polymerase III transcription termination. The x-axis values are –log_10_ of raw *p* values

**Fig. 4:**
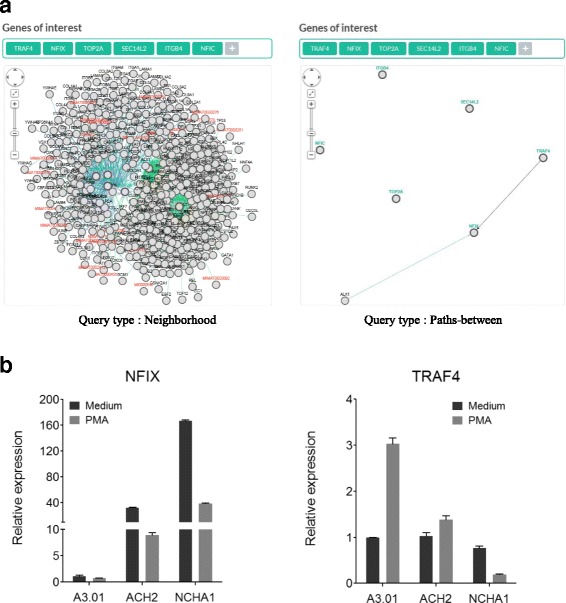
Identification of the interaction of *NFIX* with *TRAF4* in HIV-1 latently infected cells. **a** A visualization of the interactions of *TOP2A, ITGB2, TRAF4*, and *SEC14L2* with *NFIC* and *NFIX* by PCViz (Pathway Commons Network Visualizer). The query type can control the size of the network or interactions by filtering genes. The neighborhood in query type shows the overall frequency of alteration for each gene in the network and the paths between in query type shows the direct interaction of each gene in the network. **b** The result of real-time PCR in treatment with/without PMA in HIV-1 latently infected cells. Cells were treated with PMA at a final concentration of 10 ng/ml for 24 h. All experiments were performed in triplicate. *Error bars* indicate the standard deviation

First, 38 decreased and 41 increased islands were transformed to genes corresponding to each island (Additional file 5). To investigate the functional annotation of the genes associated with the 38 decreased and 41 increased islands, gene ontology enrichment analysis was performed using the WEB-based GEne SeT AnaLysis Toolkit (WebGestalt) [19]. The 38 genes associated with decreased islands were involved in the regulation of biological process, regulation of cellular process, biological regulation, and purinergic receptor signaling pathway. The 41 genes associated with increased islands were involved in nucleic acid binding, calcium-activated cation channel activity, DNA binding, and zinc ion binding (Additional file 6). Subsequently, Pathway Commons analysis was performed to further investigate the biological pathways of genes associated with decreased and increased islands. The p63 transcription factor network was strongly linked to the 38 genes with decreased islands (Fig. 3a), and the RNA polymerase transcription termination pathway was linked to the 41 genes with increased islands (Fig. 3b). Especially, DNA topoisomerase 2 alpha (*TOP2A*), integrin beta 4 subunit (*ITGB2*), TNF receptor association factor 4 (*TRAF4*), and SEC14-like lipid binding 2 (*SEC14L2*) genes associated with decreased islands were related to the p63 transcription factor network. While, nuclear factor IC (*NFIC*) and nuclear factor IX (*NFIX*) with increased islands were linked to the RNA polymerase transcription termination pathway. To examine interactions between *TOP2A*, *ITGB2*, *TRAF4*, *SEC14L2*, *NFIC*, and *NFIX*, the Pathway Commons Network Visualizer was used (Fig. 4a). *NFIX* was found to interact with *TRAF4*, which suggests that genes related to decreased and increased islands can mutually interact in the maintenance of HIV-1 latency.

### Investigation of the interaction between NFIX and TRAF4 for HIV-1 latency

Lastly, the expression of *NFIX* and *TRAF4* was examined using real-time PCR between HIV-1 latently infected cell lines, ACH2 and NCHA1, and an uninfected cell line, A3.01 (Fig. 4b). *NFIX* was highly expressed in HIV-1 latently infected cell lines compared with the uninfected cell line. The expression of *TRAF4* was similar in all cell lines. After treatment by phorbol-13-myristate-12-acetate (PMA), *NFIX* showed a dramatic reduction in expression (nearly fourfold) in HIV-1 latently infected cell lines compared with untreated cells. *TRAF4* was increased threefold in only A3.01 cells but was decreased in NCHA1 cells. These results suggest that *NFIX* might affect the reactivation and maintenance of HIV-1 latency.

## Discussion

In this study, we re-analyzed ChIP-seq data obtained from GEO data repositories to investigate whether specific chromosomal distribution of histone modifications effects on HIV-1 latency and to find novel latency-related genes. As whole distribution of histone modification was unchanged before and after HIV-1 integration [10], chromatin domains which show enriched epigenetic features partially might be potential factors for the establishment or maintenance of HIV-1 latency. To elucidate the connection between chromosomal distribution of histone modification and HIV-1 latency, H3K4me3 and H3K9ac binding distribution patterns were determined using CEAS software. Chromosomes 16, 17, 19, and 22 were significantly enriched in both histone modifications of decreased and increased binding sites.

Accordingly, the alternation of chromatin conditions in host cells induced by HIV-1 infection might play an important role in viral latency. These results indicate that there may be common factors governing HIV-1 latency. Thought genome-wide analysis of histone modifications in HIV-1 latently infected cell lines, cyclin-dependent kinase inhibitor 1A (*CDKN1A*) and *cyclin D2* were given into latency-related genes [10].

Based on Pathway Commons analysis, *NFIX* from RNA polymerase transcription termination pathway and *TRAF4* from p63 transcription factor network were strongly linked to each other.

Nuclear factor one (*NFI*) is a family of transcription factors which consist of four related members, *NFIA, NFIB, NFIC*, and *NFIX* [20]. *NFIX* is known to bind to the palindromic sequence in viral and cellular promoters [21]. These binding sites may act as activators or repressors of transcription [22].

HIV-1 long terminal repeat (LTR) is comprised of U5, R, and U3 regions. The U3 region can be divided into the core promoter, enhancer, and modulatory regions [23]. The modulatory region includes the negative regulatory element (*NRE*) [22]. Schwartz et al. [24] has shown that core sequence in the *NRE*, TGATTGGC, was the binding site for *NFI* family, and the *NFI* binding on this site has negative effect on the control of HIV-1 transcription in Jurkat cells. In this study, *NFIX* showed the increased acetylation (H3K9ac) and methylation (H3K4me3) in HIV-1 latently infected cell lines. These will allow transcription factors to easily access to the promoter of *NFIX* and increase the mRNA expression level of *NFIX* in HIV-1 latently infected cells. Consequently, increased *NFIX* may bind to core sequence in the *NRE* and then repress the transcription of HIV-1. In addition, CCAAT/enhancer binding protein (*C/EBP*) was known to be necessary for HIV-1 replication [25] and HIV-1 LTR contains three *C/EBP* binding sites. *NFIX* was previously named as CCAAT-binding transcription factor. This will provide a possibility that *C/EBP* and *NFIX* may compete for binding sites of HIV-1 LTR each other. Therefore, *C/EBP* or *NFIX* binding on HIV-1 LTR will induce or repress HIV-1 transcription, respectively. Lastly, Biressi et al. [26] reported that *NFIX* is strongly expressed in the fetus and makes a complex with *PKC* theta and then activates *MEF2A*. These results suggest that *NFIX* may negatively control HIV-1 transcription via *PKC* pathway.

*TRAF4* is known to encode a member of the TNF receptor association factor (*TRAF*) family and interact with neurotrophin receptor p75 (*NTR*). Also, it negatively controls *NTR*-induced cell death and *NF-kappa B* activation [27, 28]. Meanwhile, Xu et al. [29] showed that co-expression of p47 (*phox*) and *TRAF4* increase oxidant production and c-Jun N-terminal kinase (*JNK*) activation and that HIV-1 Tat activates *JNK* via signaling pathway at the level of *TRAF4*. In contrast to *NFIX*, *TRAF4* showed the decreased (H3K9ac) and methylation (H3K4me3) on HIV-1 latency. As a result, the decreased expression of *TRAF4* may reduce activity of *JNK* to maintain HIV-1 latency. However, *TRAF* was not seen to directly control the expression of *NFIX* in our study.

## Conclusions

In conclusion, these results suggest that the unique enrichment of histone modifications and its linked genes in specific chromosomes might play a critical role in the establishment and maintenance of HIV-1 latency.

## Methods

### Cell culture

A3.01, ACH2, and NCHA1 cells were maintained in RPMI 1640 medium supplemented with 10% FBS, 5% penicillin-streptomycin, and 2 mM glutamine. For comparison of *NFIX* expression between the normal and reactivation state, PMA was used at a final concentration of 10 ng/ml for 24 h.

### ChIP-seq data analysis

To analysis of histone modifications for H3K4me3 and H3K9ac in HIV-1 latently infected cells, ChIP-seq data was obtained from GEO data repositories (GSE58246). Enriched histone islands of the genome were identified by comparing the chromatin immunoprecipitated (ChIPed) samples to the input samples using the SICER 1.1 program with the following parameters: window size = 200, gap size = 400, *E* value = 0.01, and FDR = 0.05. The overlapped histone islands were identified by hypergeometric optimization of motif enrichment (HOMER) programs [30] with maximum distance to merge (“given”). For genomic distribution of differential binding sites, CEAS program was used. Genomic position was plotted onto the chromosome map using the idiographica webtool (http://www.ncrna.org/idiographica). Gene ontology enrichment analysis was performed using WebGestalt, and interaction of genes was visualized using the Pathway Commons Network Visualizer (PCViz, http://www.pathwaycommons.org/pcviz).

### Quantitative real-time PCR

Total RNA was extracted from cells using TRIzol reagent, and 1 μg of RNA was reverse-transcribed using Superscript III cDNA synthesis kit (Invitrogen). *NFIX* and *TRAF4* quantitative reverse transcription PCR was performed on a 7500 real-time PCR system (Applied Biosystems) using SYBR Green PCR Master Mix (Applied Biosystems). The relative expression was calculated as follows using the ΔΔCt method: fold change of enrichment = 2 delta ((Ct-ChIPed)-(Ct-Input)). ΔCt values were determined using a the 7500 real-time PCR system software (Applied Biosystems) with *GAPDH* as an endogenous control. The following primers were used for real-time PCR:

- *NFIX* forward primer (5′-AGGAGATGCGGACATCAAA-3′), *NFIX* reverse primer (5′-TACTCTCACCAGCTCCGTCA-3′), *TRAF4* forward primer (5′-AGGAGTTCGTCTTTGACACCATC-3′), *TRAF4* reverse primer (5′-CTTTGAATGGGCAGAGCACC-3′), *GAPDH* forward primer (5′-GAAGGTGAAGGTCGGAGTC-3′), and *GAPDH* reverse primer (5′-GAAGATGGTGATGGGATTTC-3′).

## Additional files

Additional file 1: Chromosomal distribution of H3K4me3 binding sites in HIV-1 latently infected cells. Enrichment pattern of increased islands of H3K4me3 among individual chromosomes is shown as bar chart. Percent of total H3K4me3 islands (red bar) and what would be expected by random chance (blue bars) for each chromosome is shown. The value in parenthesis means *p* value.

Additional file 2: Chromosomal distribution of H3K9ac binding sites in HIV-1 latently infected cells. Enrichment pattern of decreased islands of H3K9ac among individual chromosomes is shown as bar chart. Percent of total H3K9ac islands (red bar) and what would be expected by random chance (blue bars) for each chromosome is shown. The value in parenthesis means *p* value.

Additional file 3: Chromosomal distribution of H3K9ac binding sites in HIV-1 latently infected cells. Enrichment pattern of increased islands of H3K9ac among individual chromosomes is shown as bar chart. Percent of total H3K9ac islands (red bar) and what would be expected by random chance (blue bars) for each chromosome is shown. The value in parenthesis means *p* value.

Additional file 4: The ratio of percentage of total H3K4me3 or H3K9ac islands to percentage expected by random chance for each chromosome in three different HIV-1 latently infected cell lines.

Additional file 5: Lists of 38 decreased genes and 41 increased genes identified in the co-occurrence of two histone modifications, H3K4me3 and H3K9ac.

Additional file 6: Functional annotation of 38 decreased (A) and 41 increased (B) genes in chromosomes 16, 17, 19, and 22 of HIV-1 latently infected cells using WebGestalt. The *x*-axis values are –log_10_ of raw *p* values.

## Abbreviations

ART: Antiretroviral therapy
cART: Combination antiretroviral therapy
*CDKN1A*: Cyclin-dependent kinase inhibitor 1A
CEAS: Cis-regulatory element annotation system
ChIPed: Chromatin immunoprecipitated
ChIP-seq: Chromatin immunoprecipitation-high throughput parallel DNA sequencing
H3K4me3: Histone 3 lysine 4 trimethylated
H3K9ac: Histone 3 lysine 9 acetylated
HOMER: Hypergeometric optimization of motif enrichment
*ITGB2*: Integrin beta 4 subunit
*NFIX*: Nuclear factor IX
PMA: Phorbol-13-myristate-12-acetate
*SEC14L2*: SEC14-like lipid binding 2
SICER: Spatial clustering for identification of ChIP-enriched regions
*TOP2A*: DNA topoisomerase 2 alpha
*TRAF4*: TNF receptor association factor 4
WebGestalt: WEB-based GEne SeT AnaLysis Toolkit
