# Supplementary material for: Identification of novel genes associated with HIV-1 latency by analysis of histone modifications
Source: Hum Genomics. 2017 May 12;11:9. doi: 10.1186/s40246-017-0105-7 (PMC5429561; doi:10.1186/s40246-017-0105-7)
Supplement: Supplementary file 3 — Chromosomal distribution of H3K9ac binding sites in HIV-1 latently infected cells. Enrichment pattern of increased islands of H3K9ac among individual chromosomes is shown as bar chart. Percent of total H3K9ac islands (red bar) and what would be expected by random chance (blue bars) for each chromosome is shown. The value in parenthesis means p value. [file 40246_2017_105_MOESM3_ESM.pptx]

## Slide 1
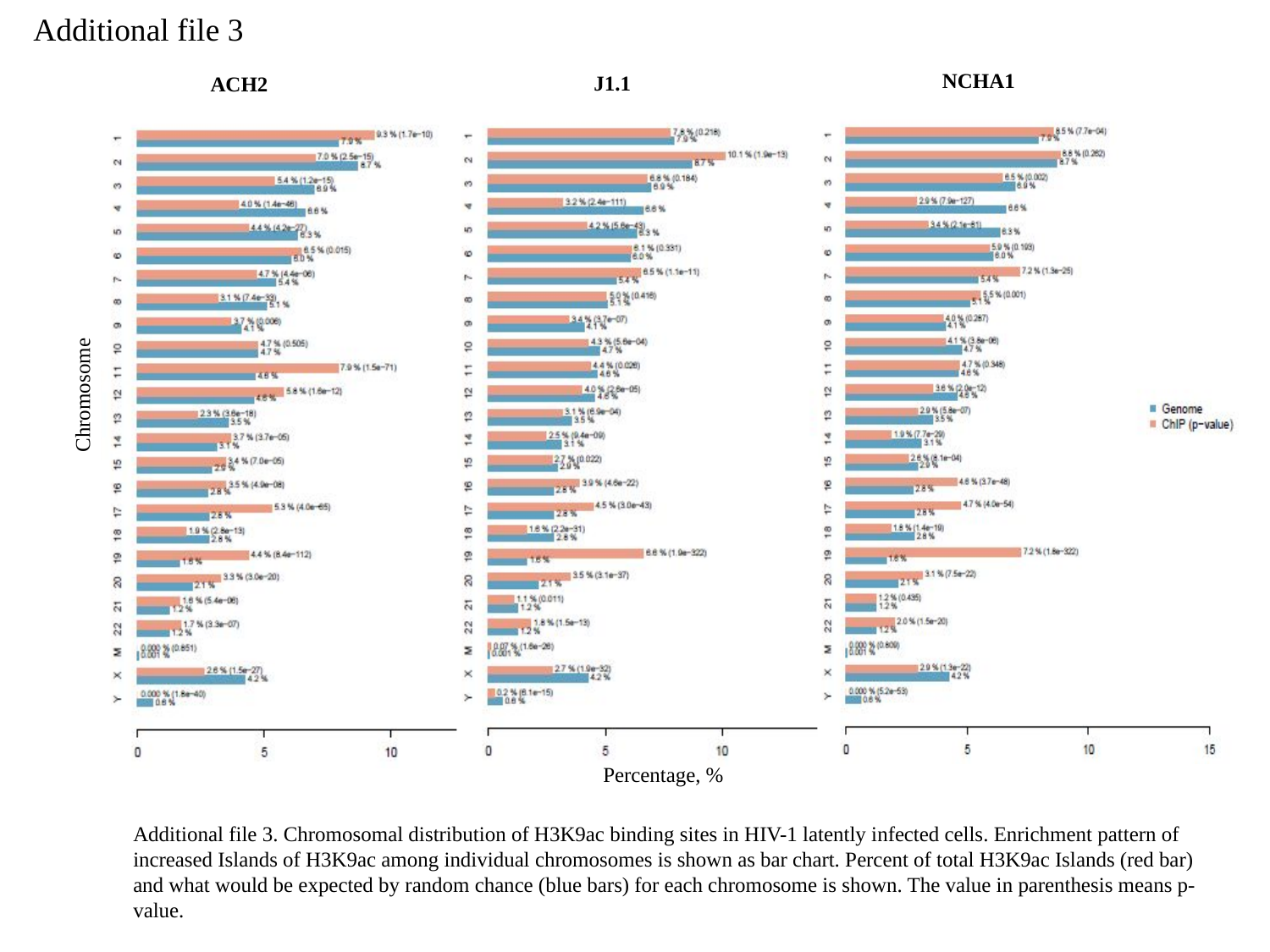

Additional file 3
NCHA1
J1.1
ACH2
Chromosome
Percentage, %
Additional file 3. Chromosomal distribution of H3K9ac binding sites in HIV-1 latently infected cells. Enrichment pattern of increased Islands of H3K9ac among individual chromosomes is shown as bar chart. Percent of total H3K9ac Islands (red bar) and what would be expected by random chance (blue bars) for each chromosome is shown. The value in parenthesis means p-value.
