# Supplementary material for: Identification of novel genes associated with HIV-1 latency by analysis of histone modifications
Source: Hum Genomics. 2017 May 12;11:9. doi: 10.1186/s40246-017-0105-7 (PMC5429561; doi:10.1186/s40246-017-0105-7)
Supplement: Supplementary file 4 — The ratio of percentage of total H3K4me3 or H3K9ac islands to percentage expected by random chance for each chromosome in three different HIV-1 latently infected cell lines. [file 40246_2017_105_MOESM4_ESM.docx]

**Additional file 4. The ratio of percentage of total H3K4me3 or H3K9ac islands to percentage expected by random chance for each chromosome in three different HIV-1 latently infected cell lines.**

| **H3K4me3_Dec** | | | | | |
| --- | --- | --- | --- | --- | --- |
| Chr (R) | ACH2 | J1.1 | NCHA1 | Ave | Ratio (Ave/R) |
| Chr1 (7.9) | 10.9 | 12.6 | 12.2 | 11.9 | 1.5 |
| Chr12 (4.6) | 5.7 | 6.1 | 7.3 | 6.4 | 1.4 |
| Chr16 (2.8)^†^ | 3.6 | 3.6 | 3.8 | 3.7 | 1.3 |
| Chr17 (2.8)^†^ | 6.2 | 6.8 | 8 | 7 | 2.5 |
| Chr19 (1.6)^†^ | 4.4 | 3 | 3 | 3.5 | 2.2 |
| Chr22 (1.2)^†^ | 2.8 | 2.9 | 2.9 | 2.9 | 2.4 |
| **H3K4me3_Inc** | | | | | |
| Chr (R) | ACH2 | J1.1 | NCHA1 | Ave | Ratio (Ave/R) |
| Chr16 (2.8) | 5.4 | 4.6 | 4.4 | 4.8 | 1.7 |
| Chr17 (2.8) | 5.4 | 4.7 | 3.8 | 4.6 | 1.7 |
| Chr19 (1.6) | 5.9 | 9.2 | 8.2 | 7.8 | 4.9 |
| Chr20 (2.1) | 3.5 | 3 | 2.3 | 2.9 | 1.4 |
| Chr22 (1.2) | 2.2 | 1.9 | 2 | 2 | 1.7 |
| **H3K9ac_Dec** | | | | | |
| Chr (R) | ACH2 | J1.1 | NCHA1 | Ave | Ratio (Ave/R) |
| Chr1 (7.9) | 11.6 | 11.2 | 11.6 | 11.5 | 1.5 |
| Chr11 (4.6) | 5.3 | 6 | 6.4 | 5.9 | 1.3 |
| Chr14 (3.1) | 4.1 | 3.9 | 4.9 | 4.3 | 1.4 |
| Chr16 (2.8) | 5.8 | 4.3 | 5.2 | 5.1 | 1.8 |
| Chr17 (2.8) | 7.6 | 7 | 7.8 | 7.5 | 2.7 |
| Chr19 (1.6) | 7 | 4.7 | 5 | 5.6 | 3.5 |
| Chr20 (2.1) | 2.5 | 2.7 | 3.5 | 2.9 | 1.4 |
| Chr22 (1.2) | 3.9 | 2.6 | 3.1 | 3.2 | 2.7 |
| **H3K9ac_Inc** | | | | | |
| Chr (R) | ACH2 | J1.1 | NCHA1 | Ave | Ratio (Ave/R) |
| Chr16 (2.8) | 3.5 | 3.9 | 4.6 | 4 | 1.4 |
| Chr17 (2.8) | 5.3 | 4.5 | 4.7 | 4.8 | 1.7 |
| Chr19 (1.6) | 4.4 | 6.6 | 7.2 | 6.1 | 3.8 |
| Chr20 (2.1) | 3.3 | 3.5 | 3.1 | 3.3 | 1.6 |
| Chr22 (1.2) | 1.7 | 1.8 | 2 | 1.8 | 1.5 |

Chr: Chromosome, R: The percentage of binding sites expected by random chance, Ave: average of the percentage of total H3K4me3 or H3K9ac islands in three different HIV-1 latently infected cell lines. Chromosomes were selected in above 1.3 ratio among three HIV-1 latently infected cell lines. †These chromosomes exist in common at H3K4me3_Dec, H3K4me3_Inc, H3K9ac_Dec and H3K9ac_Inc.
