# Supplementary material for: Identification of novel genes associated with HIV-1 latency by analysis of histone modifications
Source: Hum Genomics. 2017 May 12;11:9. doi: 10.1186/s40246-017-0105-7 (PMC5429561; doi:10.1186/s40246-017-0105-7)
Supplement: Supplementary file 5 — Lists of 38 decreased genes and 41 increased genes identified in the co-occurrence of two histone modifications, H3K4me3 and H3K9ac. [file 40246_2017_105_MOESM5_ESM.docx]

**Additional file 5. Lists of 38 decreased genes and 41 increased genes identified in the co-occurrence of two histone modifications, H3K4me3 and H3K9ac.**

| Dec (38 genes) | | | |
| --- | --- | --- | --- |
| AKTIP | C17ORF53 | CANT1 | CBX1 |
| CD7 | CELSR1 | CNN2 | DLG4 |
| DYRK1B | EPOR | GGN | HAS3 |
| IGLL1 | ITGB4 | LAT | LMF2 |
| LOC100129697 | LRFN1 | METTL22 | MIR301B |
| NLK | NT5C3B | P2RX5 | PHF23 |
| PPM1D | PRPSAP2 | RAC2 | RANGAP1 |
| RANGRF | SEC14L2 | SMIM24 | SPAG5 |
| TAB1 | TEN1-CDK3 | TNFSF12 | TOP2A |
| TRAF4 | ZBTB4 |  |  |
| Inc (41 genes) | | | |
| C16ORF92 | C17ORF97 | C19ORF60 | CARD8 |
| CBX9 | CPNE2 | CYB561 | DNASE2 |
| EFCAB13 | ENKD1 | HSH2D | KCNN4 |
| LAIR1 (59571401^*^) | LAIR1(59576001) | LINC00910 | LIPE-AS1 |
| LOC101928855 | LSM4 | MIR4754 | MMP25 |
| MT1F | NFIC | NFIX | PRR14 |
| PTPRH | RFFL | RNF135 | RPS19 |
| 09-Sep | SLC16A5 | SREBF1 | TBX1 |
| TRPM4 | ZNF137P | ZNF253 | ZNF284 |
| ZNF296 | ZNF48 | ZNF75A | ZNF785 |
| ZNF808 |  |  |  |

* Chromosome start site for ChIP-seq
