# Supplementary material for: Identification of novel genes associated with HIV-1 latency by analysis of histone modifications
Source: Hum Genomics. 2017 May 12;11:9. doi: 10.1186/s40246-017-0105-7 (PMC5429561; doi:10.1186/s40246-017-0105-7)
Supplement: Supplementary file 6 — Functional annotation of 38 decreased (A) and 41 increased (B) genes in chromosomes 16, 17, 19, and 22 of HIV-1 latently infected cells using WebGestalt. The x-axis values are –log10 of raw p values. [file 40246_2017_105_MOESM6_ESM.pptx]

## Slide 1
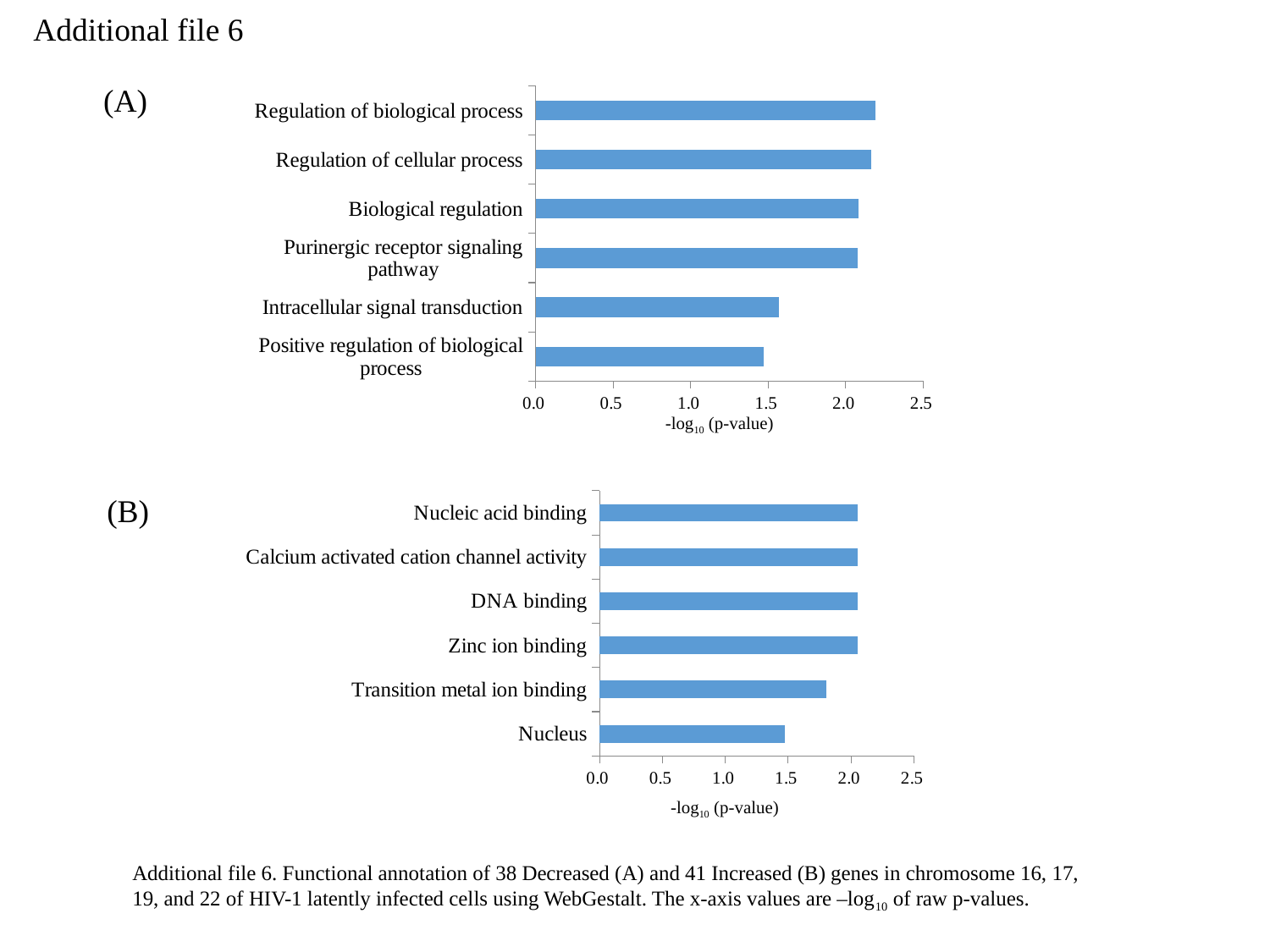

Additional file 6
(A)
### Chart
| Category | |
|---|---|
| Regulation of biological process | 2.1938200260161143 |
| Regulation of cellular process | 2.167491087293765 |
| Biological regulation | 2.080921907623928 |
| Purinergic receptor signaling pathway | 2.0757207139381184 |
| Intracellular signal transduction | 1.5686362358410126 |
| Positive regulation of biological process | 1.4710832997223446 |-log10 (p-value)
(B)
### Chart
| Category | |
|---|---|
| Nucleic acid binding | 2.0555173278498313 |
| Calcium activated cation channel activity | 2.0555173278498313 |
| DNA binding | 2.0555173278498313 |
| Zinc ion binding | 2.0555173278498313 |
| Transition metal ion binding | 1.801342913045578 |
| Nucleus | 1.4736607226101552 |-log10 (p-value)
Additional file 6. Functional annotation of 38 Decreased (A) and 41 Increased (B) genes in chromosome 16, 17, 19, and 22 of HIV-1 latently infected cells using WebGestalt. The x-axis values are –log10 of raw p-values.
